# Are overweight and obesity associated with increased risk of cesarean delivery in Mexico? A cross-sectional study from the National Survey of Health and Nutrition

**DOI:** 10.1186/s12884-019-2393-5

**Published:** 2019-07-11

**Authors:** Alexander Brenes-Monge, Biani Saavedra-Avendaño, Jacqueline Alcalde-Rabanal, Blair G. Darney

**Affiliations:** 1 Center for Evaluation Research and Surveys, National Institute of Public Health (INSP), Cuernavaca, Morelos Mexico; 2 Center for Health Systems Research, National Institute of Public Health (INSP), Cuernavaca, Morelos Mexico; 3 Center for Population Health Research, National Institute of Public Health (INSP), Cuernavaca, Morelos Mexico; 4 Department of Obstetrics and Gynecology, Oregon Health and Science University, Portland, OR USA

**Keywords:** Body mass index, Overweight, Obesity, Pregnancy, Mode of delivery, Cesarean delivery

## Abstract

**Background:**

In Mexico, obesity is a major public health problem; 71% of adults are overweight or obese. The proportion of deliveries by cesarean is also very high (45%). Women of reproductive age with overweight or obesity may be at higher risk of cesarean.

**Methods:**

We conducted a cross-sectional study to test the association between overweight and obesity (using body mass index, BMI) and cesarean delivery in Mexico using data from the 2012 National Survey of Health and Nutrition (ENSANUT). Our sample included women of reproductive age at the time of survey who reported a live birth between 2006 and 2012. We used bivariate statistics and a multivariate logistic regression model to test the association between measured BMI and self-reported cesarean delivery. We included individual, clinical, and household level confounders and used survey weights to produce population estimates.

**Results:**

Our sample consisted of 4,570 women (population *N* = 7,447,541). Overall, 44% of the women reported a cesarean at last delivery. We found differences in the proportion of cesarean delivery by BMI group (normal = 39%; 95% CI [35–43]; overweight = 42%; 95% CI [38–45]; obesity = 52%; 95% CI [48–57]; *p* < 0.001). In multivariable models controlling for socio-demographic and clinical characteristics, we found a strong and independent association between obesity and cesarean delivery among multiparous women, compared with multiparous women with normal BMI (obesity aOR: 1.60; 95% CI [1.21–2.12]).

**Conclusions:**

We provide new evidence about the proportion of women with overweight and obesity who deliver in Mexico. Multiparous women with obesity are at higher risk of cesarean delivery in Mexico than multiparous women with normal body mass index. Given the high prevalence of both obesity and cesarean delivery in Mexico, this relationship is salient for women, health care providers, and the health system.

Efforts to reduce the cesarean deliveries rate need to take the obesity epidemic into account.

**Electronic supplementary material:**

The online version of this article (10.1186/s12884-019-2393-5) contains supplementary material, which is available to authorized users.

## Introduction

In the last 25 years, the prevalence of obesity has doubled worldwide [1]; in Mexico, 71% of adults are overweight or obese, with higher proportions in women than in men [2]. Overweight and obesity prevalence has also risen among pregnant women in Mexico, more than doubling in the past 30 years [3], which could increase the risk of obstetric and perinatal complications, compared to women with a normal Body Mass Index (BMI) [4–11].

Several studies have documented positive associations between overweight and/or obesity and complications during pregnancy and delivery, such as hypertensive disorders [12–14] and gestational diabetes [15–17]; in US populations those occur most frequently in pregnant women of Hispanic descent [18]. Increased time of labor [19–24], dystocia, and increased risk of cesarean delivery (CD) have also been found to be associated with overweight/obesity [25–27]. It is thus important to examine mode of delivery in the context of complications associated with obesity.

In Mexico, the overwhelming majority (94%) of women deliver in health facilities, and the proportion of deliveries via cesarean reached 45% of all births in 2012 [28]. This proportion of CD is considered high relative to the World Health Organization’s (WHO) target cesarean rate of 10–15% of all births [29]. In addition to clinical indications for CD, preferences of both women and physicians influence the use of CD, which suggests that some cesareans are performed in Mexico without following established clinical criteria [28].

The relationship of BMI and cesarean delivery had been studied previously; a meta-analysis reported a positive association between cesarean delivery and overweight, obese, and severely obese women [30]. However, studies of mode of delivery in Mexico have focused on receipt of antenatal care and socio-demographic differences [31]; we have no data about the relationship of overweight and obesity and CD.

Given the high prevalence of both overweight/obesity and CD in Mexico, this is a salient issue for women, health care providers, and the health system. The aim of this study was to determine the relationship between overweight/obesity (measured using BMI) and cesarean delivery in Mexican women. We hypothesized that overweight/obesity would be independently associated with CD in the Mexican population.

## Methods

### Data and sample

**Fig. 1:**
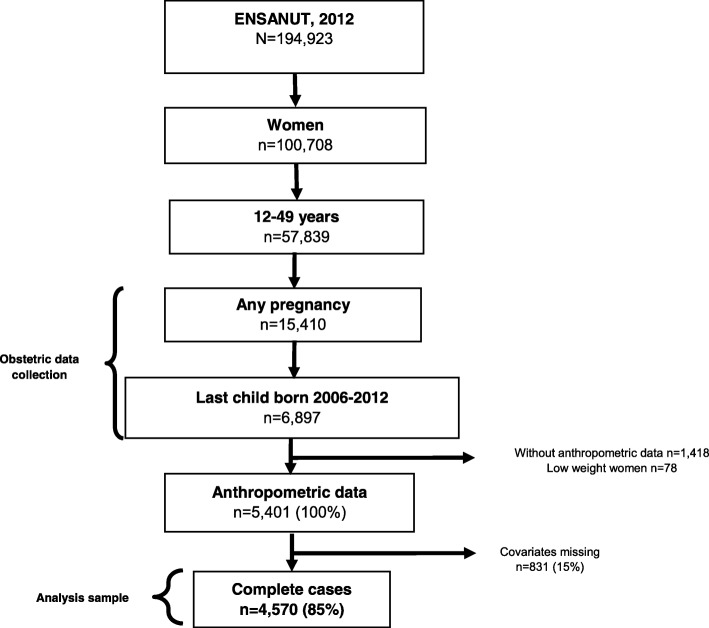
Flow diagram of analysis sample selection

We conducted a cross-sectional study using data from the 2012 National Survey of Health and Nutrition (Encuesta Nacional de Salud y Nutricion, ENSANUT); the 2016 ENSANUT does not include information about delivery so these are the most recent data available. The survey uses probabilistic sampling and is representative at the state level and urban and rural strata. It is conducted approximately every 5 years through face to face interviews and includes anthropometric measurements of a subsample [2]. The ENSANUT survey collects written informed consent from all participants and only de-identified data are publically available. (Detailed information regarding ENSANUT is available at http://ensanut.insp.mx/). The ENSANUT includes a reproductive health service utilization module asked only of women 12–49 years old at time of survey who reported a live birth in the 5 years preceding the survey (between 2006 and 2012; Fig. 1). Our analytic sample included women with reproductive health data and with complete data of anthropometric measurements of weight and height, (85% had complete information; Fig. 1).

Our dependent variable was self-reported mode of delivery of the last pregnancy (vaginal delivery or CD); CD included both planned and emergency cesareans. Our key independent variable was BMI, calculated from data of the anthropometric measurements of weight and height, using the formula (weight (kilograms) / height (in meters^2^); adolescent mothers (< 20 years) were categorized by standard deviations of age-specific BMI [27]. Data were categorized as either normal (BMI 18.5 - ≤24.9), overweight (BMI 25–29.9), or obese (BMI ≥ 30) [28]. We collapsed BMI into commonly used categories [32, 33], which permits comparison with previous literature. Women with low weight (BMI <  20) were excluded, because we considered this group as a separate risk condition of prenatal and perinatal complications by itself (Fig. 1). [29–31], and not the focus of this study. The reference group for all analyses were women with a normal BMI.

We included several covariates related to prenatal care, comorbidities, and complications. Prenatal covariates were: parity (nulliparous and multiparous), antenatal care initiation (early -in the first trimester- or late -after the first trimester-) and the total number of prenatal consultations, categorized according to Mexican government guidelines (at least five consultations or fewer than five consultations) [32]. We included pre-existing diabetes mellitus and hypertension as well as an indicator for self-reported presence of any complications during pregnancy, including hypertension disorders, gestational diabetes, infections (urinary or sexually transmitted diseases), anemia and threatened miscarriage. We also included the self-reported presence of any complications during labor and delivery (malpresentation, obstructed labor, preterm birth, and postpartum hemorrhage). We included type of health facility where the delivery occurred (employment based insurance facilities, public insurance facilities and private facilities).

We also included several socio-demographic variables that could be associated with our exposure and/or outcome. We included woman’s age at delivery, categorized in five-year groups. We measured education by the highest level achieved, (secondary/middle school or lower, high school, and greater than high school). We included household socioeconomic status in quintiles (quintile I being the poorest). The 2012 ENSANUT household socioeconomic indicator is based on household materials and ownership of consumer goods and generated using imputation methods [33].

We included indigenous ethnicity, measured by whether anyone in the household speaks an indigenous language, [34, 35] and geographic region (northern, central, central-western, south-southeast, taken directly from the state classification used by the ENSANUT) [26]. We included health insurance, categorized as either employment-based insurance (for those who work in the formal sector), public insurance (run by the Ministry of Health for low-income people) or no coverage (women who reported no health insurance affiliation). In addition, we calculated the time elapsed between date of delivery and anthropometric measurement (date of survey), in order to be able to control for recall bias in variables as well as changes that may have occurred in BMI between delivery and the survey.

### Analysis

We first used chi-square tests to assess bivariate differences in sociodemographic and health characteristics by BMI category. We also disaggregated self-reported complications during pregnancy and delivery and used chi-square to test for differences in individual complications by BMI. We also used chi-square tests to assess differences in our main outcome (mode of delivery) by BMI. We used survey weights in all analyses to account for the complex sampling scheme of the ENSANUT and produce population estimates.

We compared demographic, health characteristics between our analytic sample and those women excluded due to missing data, and there were no differences in our outcome and in most covariates, an additional file shows this in more detail [see Additional file 1]. Women excluded due to missing data were more likely to reside in indigenous households and in the central-western and south-southeast region, and more likely to deliver in public and private facilities (versus employment-based facilities).

We first ran a bivariate model including only BMI and CD, stratified by parity (nulliparous vs multiparous). We then developed a multivariate logistic regression model stratified by parity, to test the association between mode of delivery and BMI.

Covariates included in the final logistic model were age at delivery, education level, socioeconomic status, indigenous ethnicity, initiation of prenatal care, antenatal consultations, diabetes mellitus, hypertension, complications during pregnancy grouped, complications at delivery grouped, place of delivery, and health insurance.

We also performed a sensitivity analysis stratifying by the time elapsed between date of delivery and anthropometric measurement (less than 2 years, two-four years, more than 4 years) according to parity. The interpretation of our findings was similar, suggesting that recall bias was not a problem; an additional file shows this in more detail [see Additional files 2 & 3]. We therefore simply included elapsed time between delivery and BMI measurement as a control variable in our final regression model. We ran several models to compare including complications as a dichotomous variable (presence of any complication yes or no), then we disaggregated the complications and only included those individual complications that were significantly different by BMI in bivariate tests. Finally, we tested a model in which we only included those complications during pregnancy and delivery that are clinically related with overweight and obesity (hypertension, preeclampsia-eclampsia and diabetes mellitus). Using complications as a binary variable produced the same results and a better-fitting model so we used presence or absence of complications during pregnancy or delivery in our analysis, in addition to diabetes and hypertension.

## Results

Our analytic sample included 4,570 women between 12 and 49 years old who reported a live birth between 2006 and 2012. This sample represents a population of 7,277,541 Mexican women. Overweight and obesity prevalence of women of reproductive age was 66.2% (36.4% overweight and 29.8% obese) Table 1. Overweight and obese women were more likely to be older than normal BMI women. The distribution of socioeconomic status was similar by BMI (Table 1).

**Table 1:** Characteristics of the study population by body mass index

	Body mass index								
	Normal		Overweight		Obesity		Total		
n (%) sample	1,463 (32.01)		1,731 (37.88)		1,376 (30.01)		4,570 (100)		
n (%) survey weights	2,452,531 (33.8)		2,649,025 (36.4)		2,175,985 (29.8)		7,277,541 (100)		
	% [CI]								*P*-value
Age at delivery (years)									
*12–19*	0.27	[0.24,0.31]	0.13	[0.11,0.15]	0.10	[0.08,0.12]	0.17	[0.15,0.18]	p = 0.001
*20–24*	0.31	[0.28,0.36]	0.28	[0.24,0.32]	0.25	[0.21,0.29]	0.28	[0.26,0.30]	
*25–29*	0.24	[0.21,0.28]	0.26	[0.22,0.29]	0.26	[0.23,0.30]	0.25	[0.24,0.27]	
*30–34*	0.12	[0.10,0.15]	0.22	[0.19,0.25]	0.26	[0.22,0.29]	0.20	[0.18,0.22]	
*35–39*	0.04	[0.03,0.05]	0.10	[0.08,0.12]	0.12	[0.09,0.15]	0.08	[0.07,0.09]	
*40–44*	0.01	[0.01,0.02]	0.02	[0.01,0.03]	0.02	[0.01,0.03]	0.02	[0.01,0.02]	
*45–49*	0.00	[0.00,0.00]	0.00	[0.00,0.01]	0.00	[0.00,0.01]	0.00	[0.00,0.00]	
Educational level									
*Primary or less*	0.22	[0.19,0.25]	0.27	[0.24,0.31]	0.30	[0.26,0.33]	0.26	[0.24,0.28]	*p* = 0.019
*High school*	0.41	[0.37,0.45]	0.39	[0.36,0.43]	0.40	[0.36,0.44]	0.40	[0.38,0.42]	
*Greater than high school*	0.37	[0.33,0.41]	0.34	[0.30,0.37]	0.30	[0.26,0.35]	0.34	[0.31,0.36]	
Socioeconomic status									
*I (lower)*	0.35	[0.31,0.39]	0.33	[0.30,0.37]	0.35	[0.32,0.39]	0.35	[0.32,0.37]	*p* = 0.145
*II*	0.22	[0.18,0.25]	0.24	[0.21,0.27]	0.20	[0.17,0.23]	0.22	[0.20,0.24]	
*III*	0.17	[0.14,0.20]	0.21	[0.18,0.24]	0.16	[0.14,0.20]	0.18	[0.16,0.20]	
*IV*	0.16	[0.13,0.20]	0.14	[0.12,0.17]	0.18	[0.15,0.22]	0.16	[0.14,0.18]	
*V*	0.10	[0.08,0.13]	0.08	[0.06,0.10]	0.10	[0.07,0.14]	0.09	[0.08,0.11]	
Indigenous ethnicity	0.06	[0.04,0.07]	0.08	[0.06,0.10]	0.04	[0.03,0.06]	0.06	[0.05,0.07]	*p* = 0.007
Region									
*North*	0.21	[0.19,0.24]	0.19	[0.17,0.22]	0.23	[0.20,0.26]	0.21	[0.20,0.22]	*p* = 0.314
*Central*	0.35	[0.31,0.40]	0.33	[0.29,0.36]	0.31	[0.27,0.36]	0.33	[0.31,0.35]	
*Central-western*	0.22	[0.19,0.25]	0.22	[0.19,0.24]	0.21	[0.18,0.24]	0.22	[0.20,0.23]	
*South-southeast*	0.22	[0.19,0.25]	0.26	[0.24,0.29]	0.25	[0.22,0.28]	0.24	[0.23,0.26]	
Parity									
*Nulliparous*	0.36	[0.32,0.40]	0.24	[0.21,0.27]	0.20	[0.16,0.24]	0.27	[0.25,0.29]	p = 0.001
*Multiparous*	0.64	[0.60,0.68]	0.76	[0.73,0.79]	0.80	[0.76,0.84]	0.73	[0.71,0.75]	
Initiation of prenatal care									
Early	0.81	[0.78,0.84]	0.86	[0.83,0.88]	0.85	[0.81,0.87]	0.84	[0.82,0.85]	*p* = 0.069
Late	0.19	[0.16,0.22]	0.14	[0.12,0.17]	0.15	[0.13,0.19]	0.16	[0.15,0.18]	
Antenatal consultations									
*Five or more*	0.86	[0.83,0.88]	0.89	[0.87,0.91]	0.91	[0.88,0.93]	0.89	[0.87,0.90]	*p* = 0.010
*Less than five*	0.14	[0.12,0.17]	0.11	[0.09,0.13]	0.09	[0.07,0.12]	0.11	[0.10,0.13]	
Diabetes Mellitus	0.01	[0.00,0.01]	0.02	[0.01,0.04]	0.03	[0.02,0.04]	0.02	[0.01,0.02]	*p* = 0.013
Hypertension	0.06	[0.05,0.09]	0.07	[0.05,0.09]	0.13	[0.10,0.16]	0.08	[0.07,0.10]	p = 0.001
Complications during pregnancy	0.61	[0.57,0.64]	0.58	[0.54,0.62]	0.61	[0.57,0.65]	0.60	[0.58,0.62]	*p* = 0.442
Complications at delivery	0.20	[0.17,0.23]	0.21	[0.18,0.25]	0.24	[0.20,0.27]	0.21	[0.20,0.23]	*p* = 0.300
Place of delivery									
*Social security*	0.29	[0.25,0.33]	0.31	[0.28,0.35]	0.30	[0.26,0.34]	0.30	[0.28,0.32]	*p* = 0.659
*Public service facilities*	0.47	[0.42,0.51]	0.46	[0.42,0.49]	0.48	[0.44,0.53]	0.47	[0.44,0.49]	
*Private facilities*	0.25	[0.21,0.29]	0.23	[0.20,0.26]	0.22	[0.18,0.26]	0.23	[0.21,0.26]	
Health insurance									
*Social security*	0.27	[0.23,0.31]	0.31	[0.28,0.34]	0.29	[0.26,0.33]	0.29	[0.27,0.31]	*p* = 0.478
*Public service*	0.50	[0.46,0.55]	0.50	[0.46,0.54]	0.50	[0.46,0.54]	0.50	[0.48,0.53]	
*No coverage*	0.23	[0.19,0.27]	0.19	[0.16,0.23]	0.21	[0.17,0.25]	0.21	[0.19,0.23]	

All proportions include survey weights

Women with overweight and obesity were more likely to be multiparous compared with normal BMI (normal BMI 64%; 95% confidence interval (CI) [60–68]; overweight 76%; 95% CI [73–79]; obesity 80%; 95% CI [76–84]; *p* = 0.001; Table 1). Women with overweight and obesity were also more likely to report existing diabetes (overweight 2% [95% CI 1–4] and obesity 3% [95% CI 2–4] vs. normal BMI 1% [95% CI 0–1]; *p* = 0.013) and hypertension (normal BMI 6%; 95% CI [5–9]; overweight 7%; 95% CI [5–9] and obesity 13%; 95% CI [10–16]; *p* = 0.001).

**Fig. 2:**
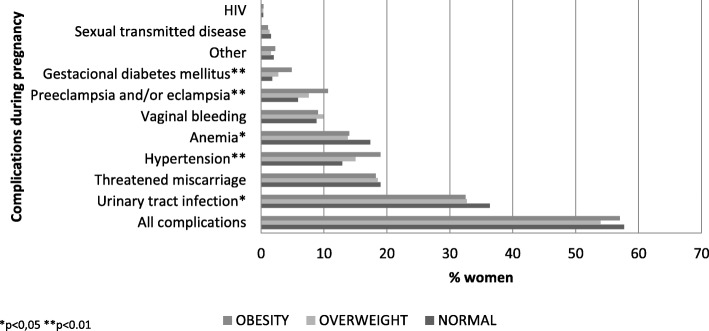
Complications during pregnancy by body mass index, Mexico, 2012

No differences in the presence of any complications (collapsed any/none) during pregnancy or delivery between BMI groups were found (normal BMI 61%; 95% CI [57–64]; overweight 58%; 95% CI [54–62], obesity 61%; 95% CI [57–65]; *p* = 0.442). When we disaggregated complications during pregnancy we found differences by BMI in hypertension (normal BMI 13%; 95% CI [10–15]; overweight 16%; 95% CI [13–18] *p* < 0.05; obesity 20%; 95% CI [17–24]; *p* = 0.004), preeclampsia-eclampsia (normal BMI 5%; 95% CI [4–8]; overweight 9%; 95% CI [7–12]; obesity 11%; 95% CI [9–14]; *p* = 0.001) and gestational diabetes mellitus (normal BMI 1%; 95% CI [1, 2]; overweight 3%; 95% CI [2–4]; obesity 5%; 95% CI [4–8]; p = 0.001). Figure 2. Complications at delivery - collapsed and disaggregated - did no show differences by BMI.

**Fig. 3:**
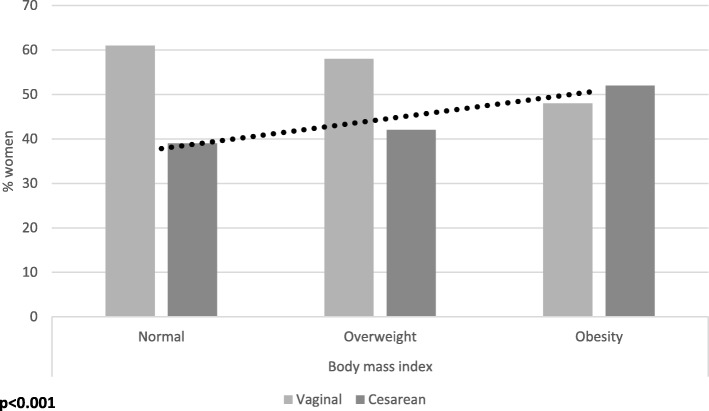
Mode of delivery by body mass index, Mexico, 2012

Women with overweight and obesity reported a proportion of CD higher than women with normal BMI (normal 39%; 95% CI [35–43]; overweight 42%; 95% CI [38–45]; obesity 52%; 95% CI [48–57]; *p* = 0.001); (Fig. 3).

Results from the bivariate model including only BMI and CD segregated by parity (nulliparous vs multiparous) show a relationship between multiparous women with obesity and CD (unadjusted odds ratio = 1.91; 95% CI [1.44–2.55]; see Additional file 4).

Our multivariable logistic regression model stratified by parity (nulliparous vs multiparous) show that multiparous women with obesity had higher odds of CD compared to women with normal BMI after controlling for confonders (adjusted odds ratio (aOR) = 1.89; 95% CI [1.36–2.63]; Table 2.). The presence of complications during delivery was also associated with CD, in both nulliparous (aOR = 3.50; 95% CI [2.06–5.94]) and multiparous women (aOR = 4.01; 95% CI [3.01–5.35]). In addition, both nulliparous and multiparous women who delivered at private facilities had higher odds of CD compared with those who delivered at social security facilities (nulliparous aOR = 1.95; 95% CI [1.08–3.53]; multiparous aOR = 2.51; 95% CI [1.72–3.68].

**Table 2:** Association between body mass index (BMI) and cesarean deliveries according to parity, Mexico, 2012

	Primiparous			Multiparous		
Sample n (%)	1,093 (23.9)			3,477 (76.1)		
Population estimates N (%)	1,943,104 (26.7)			5,334,438 (73.3)		
	aOR	[95% CI]		aOR	[95% CI]	
Body mass index (ref.: normal)						
Overweight	0.63	0.39	1.00	1.31	0.98	1.74
Obesity	1.30	0.76	2.23	1.89	1.36	2.63
Late antenatal care initiation	1.10	0.63	1.92	1.08	0.78	1.49
Five or more antenatal consultations	0.52	0.29	0.95	0.74	0.49	1.10
Complications during pregnancy	0.92	0.60	1.40	1.17	0.92	1.50
Complications at delivery	3.50	2.06	5.94	4.01	3.01	5.35
Diabetes Mellitus	0.50	0.12	2.10	1.47	0.61	3.54
Hypertension	1.56	0.69	3.50	1.02	0.67	1.57
Place of delivery (ref.: social security)						
*Public service facilities*	0.89	0.54	1.46	1.09	0.76	1.57
*Private facilities*	1.95	1.08	3.53	2.51	1.72	3.68
Age at delivery (ref.: 12–14 years)						
15–19	0.83	0.26	2.60	6.07	1.11	33.10
20–24	1.19	0.38	3.73	8.64	1.64	45.55
25–29	2.40	0.67	8.58	9.81	1.87	51.49
30–34	1.66	0.37	7.55	13.05	2.49	68.25
35–40	11.94	1.95	73.12	12.90	2.47	67.41
40–44	3.36	0.30	38.16	14.14	2.54	78.60
+ 45	–	–	–	–	–	–
Educational level (ref.: primary or less)						
*High school*	0.72	0.41	1.27	1.22	0.94	1.59
*Greater than high school*	0.73	0.39	1.37	1.61	1.16	2.23
Socioeconomic status (ref.: I quintile, lower)						
*II*	0.89	0.50	1.58	1.30	0.96	1.77
*III*	0.76	0.43	1.34	1.68	1.19	2.38
*IV*	1.07	0.57	2.00	1.33	0.86	2.04
*V*	1.14	0.53	2.42	1.89	1.10	3.27
Indigenous ethnicity	0.84	0.41	1.71	0.68	0.42	1.10
Region (ref.: north)						
*Central*	1.21	0.71	2.05	1.42	0.99	2.03
*Central-western*	1.36	0.75	2.45	1.21	0.90	1.63
*South-southeast*	2.04	1.17	3.57	1.06	0.75	1.52
Health insurance (ref.: social security)						
*Public service*	0.73	0.44	1.22	0.71	0.51	1.01
*No coverage*	0.92	0.50	1.67	0.95	0.62	1.45
Time elapsed between delivery and BMI measurement (ref.: < 2 years)						
2–4	0.99	0.63	1.57	0.95	0.68	1.31
> 4	1.22	0.75	1.98	0.92	0.67	1.26

## Discussion

We found that in Mexico, maternal overweight and obesity are common, as is cesarean delivery, and furthermore, that obesity is independently associated with cesarean delivery in multiparous women, controlling for sociodemographic and clinical characteristics that are associated with BMI and CD. Just over 42% of women with overweight and 52% of women with obesity had a CD, compared with 39% of women with normal BMI, and 1.89 higher odds of CD were found in multiparous women with obesity when compared with normal BMI women, controlling for socio-demographic and health factors. Our results support our hypothesis that in Mexico, high BMI (obesity) is independently associated with CD, controlling for socio-demographic and health characteristics.

Overall, the proportion of deliveries via CD in our study was 44%, which supports previous evidence in Mexico [28], and is comparable with CD rates in Brazil (45.9%), the Dominican Republic (41.9%) and Cuba (35.6%) [34], positioning Latin America and the Caribbean region with the highest CD rates worldwide [35, 36]. A recent study reported better maternal and neonatal outcomes when CD rates do not exceed 19% of all births [37]; the proportion of CD found in our study is three times higher than the WHO recommendation of CD [29].

Studies in Latin American countries have reported that high CD rates were positively associated with severe maternal morbidity and mortality and higher fetal mortality rates [38]. Furthermore, increases in CD rates is not associated with improvements in other perinatal outcomes [39]. Results from a meta-analysis including cohort studies of pregnant women with anthropometric measurements shows higher odds of cesarean in women with obesity, consistent with our results [40]. A Canadian study that compared maternal outcomes between women with normal BMI and extreme obesity also found a positive association between extreme obesity and CD [41].

Studies in nulliparous women have reported a positive relationship between a high BMI (overweight and obesity) and CD [42–44], which we did not find in our study. However, a study focused on the risk of caesarean section in obese women analysed by parity reported highest CD rates in multiparous women associated with a high rate of repeat elective CD [45]. This finding could be related to a policy of scheduling pre-labour CD for all women with one previous scar without attempting a trial of labour [46].

We found women with any self-reported complications at delivery had higher adjusted odds of CD compared with women who did not report any complications.

The same positive association between high BMI and cesarean was found in multiparous women without complications, as reported in previous studies [47].

Pre-pregnancy BMI has been studied previously in Mexico, focused on pregnancy, fetal and neonatal complications as the primary outcome [48–50]. This previous literature has not focused on mode of delivery as an outcome. Our study fills a gap in the literature, especially given the concurrent epidemics of obesity and CD in Mexico. Although overweight and obesity are not defined as a CD indication in clinical practice guidelines [51], our results shows a strong and independent association between high BMI and CD in multiparous women. Our findings suggest that the high CD rate and obesity epidemic are linked. Health care providers should consider BMI as important factor in maternal interventions to reduce negative maternal and perinatal outcomes [47, 52].

The Mexican Health System has recognized the importance of reducing cesareans and has created official standards, technical guidelines and clinical practice guidelines to regulate and reduce the CD rate [51]. Despite these efforts, implementation of these recommendations has faced challenges and high rates persist as reported here and previously [53–55]. Contributing factors may be the lack of training of health professionals, resistance to change and maintenance of practices not based on evidence, a perception of CD as harmless, quick and effective by pregnant women and physicians [56, 57]. Increased demand of CD by women from poor families, following trends documented in rich people, have also been reported [52].

The most important limitation of study is the timing of BMI measurement; we do not have pre-pregnancy BMI measurements, rather we have BMI measurments following delivery, at the time of survey. However, we use a large nationally representative sample with anthropometric BMI measurement (not self-report, which was previously reported to have an important bias, particularly in women [58, 59]). We explored potential bias introduced by time elapsed between delivery and survey through stratified analyses (Additional files 2 & 3) and results were robust. We provide the first population-level assessment of BMI and CD in Mexico. Our outcome and covariates (except our exposure, BMI) are self-report, which could potentially affect our results so we performed multiple sensitivity analyses with variables sensitive to recall bias (such as complications during delivery) to ensure our results were robust to model specification. Our study included only those women with complete anthropometric data, and women excluded were more vulnerable than those with complete data. The missing variables in the women excluded from the study sample corresponded to those of self-report related to sociodemographic characteristics and health history (Additional file 1). There was no lack of data in the BMI exposure variable and the type of delivery outcome. When analyzing the differences between women included and excluded from the study sample, no differences were found in the BMI exposure variable and the type of delivery outcome. Women with missing data could be those with greater vulnerability, which could limit the generalization of study results. Future studies should consider exploring in depth if these differences could be affecting the health outcome in the most vulnerable women.

## Conclusion

The results of our study provide evidence in the Mexican population of the relationship between high BMI and risk of CD. Our results further offer new evidence about the proportion of women with overweight and obesity who deliver in Mexico, which can be used to inform maternal health programs. Given the high prevalence of both obesity and CD in Mexico, this relationship is salient for women, health care providers, and the health system. Efforts to reduce the CD rate need to take the obesity epidemic into account.

## Additional files

Additional file 1: **Table S1.** Characteristics of women excluded vs. included of analysis sample, Mexico, 2012. (DOCX 14 kb)

Additional file 2: **Table S2.** Association between body mass index and cesarean delivery in nulliparous women, by time elapsed between delivery and BMI measurement, Mexico, 2012. (DOCX 16 kb)

Additional file 3: **Table S3.** Association between body mass index and cesarean delivery in multiparous women, by time elapsed between delivery and BMI measurement, Mexico, 2012. (DOCX 16 kb)

Additional file 4: **Table S4.** Association between body mass index (BMI) and cesarean deliveries according to parity, Mexico, 2012. (Unadjusted odds ratio). (DOCX 12 kb)

## Data Availability

The datasets generated during and/or analysed during the current study are available in the ENSANUT repository, [https://ensanut.insp.mx/index.php]. To access the Data Bases and Documentation section of the ENSANUT, a registration form must be complete. Subsequently, a username and password to access will be delivered. [2].

## Abbreviations

aOR: adjusted odds ratio
BIC: Bayesian Information Criterion
BMI: Body mass index
CD: Cesarean delivery
CI: confidence interval
ENSANUT: Encuesta Nacional de Salud y Nutricion (National Survey of Health and Nutrition)
WHO: World Health Organization
