# Supplementary material for: Are overweight and obesity associated with increased risk of cesarean delivery in Mexico? A cross-sectional study from the National Survey of Health and Nutrition
Source: BMC Pregnancy Childbirth. 2019 Jul 11;19:239. doi: 10.1186/s12884-019-2393-5 (PMC6624890; doi:10.1186/s12884-019-2393-5)
Supplement: Supplementary file 1 — Table S1. Characteristics of women excluded vs. included of analysis sample, Mexico, 2012. (DOCX 14 kb) [file 12884_2019_2393_MOESM1_ESM.docx]

**Results: Additional file 1**

| **Additional table 1. Characteristics of women excluded vs. included of analysis sample, Mexico, 2012.** | | | |
| --- | --- | --- | --- |
|  | **Excluded** | **Included** | **Total** |
| **n (%)** | **805 (14.98)** | **4,570 (85.02)** | **5,375 (100)** |
|  | **%** | | |
| **Body mass index** |  |  |  |
| Normal | 34.0 | 34.0 | 34.0 |
| Overweight | 38.0 | 36.0 | 37.0 |
| Obesity | 28.0 | 30.0 | 30.0 |
| **Mode of delivery** |  |  |  |
| Vaginal | 56.0 | 56.0 | 56.0 |
| Cesarean | 44.0 | 44.0 | 44.0 |
| **Primiparous** | 26.0 | 27.0 | 27.0 |
| **Late antenatal care initiation** | 17.0 | 16.0 | 16.0 |
| **5 or more antenatal consultations** | 89.0 | 89.0 | 89.0 |
| **Any complications during pregnancy** | 56.0 | 60.0 | 59.0 |
| **Place of delivery*** |  |  |  |
| Social security facilities | 29.0 | 30.0 | 30.0 |
| Public service facilities | 38.0 | 47.0 | 46.0 |
| Private service facilities | 32.0 | 23.0 | 25.0 |
| **Complications at delivery** | 17.0 | 21.0 | 21.0 |
| **Diabetes mellitus** | 2.0 | 2.0 | 2.0 |
| **Hypertension** | 7.0 | 8.0 | 8.0 |
| **Age at delivery** |  |  |  |
| 12-19 | 16.0 | 17.0 | 16.0 |
| 20-29 | 54.0 | 53.0 | 54.0 |
| 30-39 | 28.0 | 28.0 | 28.0 |
| 40-49 | 2.0 | 2.0 | 2.0 |
| **Educational level** |  |  |  |
| Primary or less | 31.0 | 26.0 | 27.0 |
| High school | 31.0 | 40.0 | 39.0 |
| Greater than high school | 38.0 | 34.0 | 34.0 |
| **Socioeconomic status** |  |  |  |
| I | 35.0 | 35.0 | 35.0 |
| II | 18.0 | 22.0 | 21.0 |
| III | 17.0 | 18.0 | 18.0 |
| IV | 17.0 | 16.0 | 16.0 |
| V | 12.0 | 9.0 | 10.0 |
| **Indigenous ethnicity**** | 11.0 | 6.0 | 7.0 |
| **Region**** |  |  |  |
| North | 18.0 | 21.0 | 21.0 |
| Central | 25.0 | 33.0 | 32.0 |
| Central-western | 32.0 | 22.0 | 23.0 |
| South-southeast | 25.0 | 24.0 | 24.0 |
| **Health insurance** |  |  |  |
| Social security | 34.0 | 29.0 | 30.0 |
| Public services | 47.0 | 50.0 | 50.0 |
| No coverage | 19.0 | 21.0 | 21.0 |
| **** p<0.01, * p<0.05** |  |  |  |
