# Supplementary material for: Are overweight and obesity associated with increased risk of cesarean delivery in Mexico? A cross-sectional study from the National Survey of Health and Nutrition
Source: BMC Pregnancy Childbirth. 2019 Jul 11;19:239. doi: 10.1186/s12884-019-2393-5 (PMC6624890; doi:10.1186/s12884-019-2393-5)
Supplement: Supplementary file 2 — Table S2. Association between body mass index and cesarean delivery in nulliparous women, by time elapsed between delivery and BMI measurement, Mexico, 2012. (DOCX 16 kb) [file 12884_2019_2393_MOESM2_ESM.docx]

| **Additional table 2. Association between body mass index and cesarean delivery in nulliparous women, by time elapsed between delivery and BMI measurement, Mexico, 2012.** | | | | | | | | | | | |
| --- | --- | --- | --- | --- | --- | --- | --- | --- | --- | --- | --- |
|  | **< 2 years** | | |  | **2 - 4 years** | | |  | **> 4 years** | | |
|  | **aOR** | **[95% CI]** | |  | **aOR** | **[95% CI]** | |  | **aOR** | **[95% CI]** | |
| **Body mass index (ref.: normal)** |  |  |  |  |  |  |  |  |  |  |  |
| Overweight | 0.92 | 0.64 | 1.32 |  | 0.76 | 0.55 | 1.05 |  | 0.92 | 0.66 | 1.28 |
| Obesity | 1.85 | 1.22 | 2.82 |  | 1.23 | 0.84 | 1.79 |  | 1.39 | 0.97 | 2.00 |
| **Late antenatal care initiation** | 1.41 | 0.90 | 2.19 |  | 0.93 | 0.62 | 1.40 |  | 1.19 | 0.80 | 1.78 |
| **Five or more antenatal consultations** | 0.57 | 0.34 | 0.96 |  | 0.65 | 0.40 | 1.04 |  | 0.60 | 0.40 | 0.92 |
| **Complications during pregnancy** | 1.12 | 0.78 | 1.59 |  | 1.05 | 0.78 | 1.41 |  | 0.91 | 0.69 | 1.20 |
| **Complications at delivery** | 3.19 | 2.10 | 4.85 |  | 3.77 | 2.70 | 5.27 |  | 4.08 | 2.86 | 5.82 |
| **Diabetes Mellitus** | 1.60 | 0.40 | 6.36 |  | 1.02 | 0.39 | 2.70 |  | 0.69 | 0.24 | 1.97 |
| **Hypertension** | 1.30 | 0.69 | 2.45 |  | 1.41 | 0.78 | 2.54 |  | 0.96 | 0.57 | 1.61 |
| **Place of delivery (ref.: social security)** |  |  |  |  |  |  |  |  |  |  |  |
| *Public service facilities* | 0.95 | 0.61 | 1.46 |  | 1.00 | 0.69 | 1.44 |  | 0.95 | 0.66 | 1.38 |
| *Private facilities* | 2.00 | 1.23 | 3.26 |  | 2.57 | 1.71 | 3.86 |  | 1.73 | 1.17 | 2.55 |
| **Age at delivery (ref.: 12 - 19 years)** |  |  |  |  |  |  |  |  |  |  |  |
| 15-19 | 0.66 | 0.22 | 1.97 |  | 0.76 | 0.24 | 2.41 |  | 0.76 | 0.22 | 2.59 |
| 20-24 | 0.89 | 0.30 | 2.66 |  | 0.95 | 0.30 | 2.99 |  | 0.99 | 0.29 | 3.31 |
| 25-29 | 0.99 | 0.32 | 3.08 |  | 1.38 | 0.43 | 4.46 |  | 1.09 | 0.32 | 3.74 |
| 30-34 | 1.11 | 0.34 | 3.66 |  | 1.33 | 0.40 | 4.40 |  | 1.25 | 0.36 | 4.33 |
| 35-40 | 1.77 | 0.50 | 6.29 |  | 1.62 | 0.45 | 5.77 |  | 1.50 | 0.42 | 5.36 |
| 40-44 | 0.93 | 0.17 | 5.02 |  | 1.88 | 0.42 | 8.39 |  | 1.65 | 0.41 | 6.61 |
| +45 | 1.00 |  |  |  | 0.15 | 0.02 | 1.27 |  | 1.00 |  |  |
| **Educational level (ref.: primary or less)** |  |  |  |  |  |  |  |  |  |  |  |
| *High school* | 0.97 | 0.62 | 1.51 |  | 1.20 | 0.82 | 1.76 |  | 1.00 | 0.74 | 1.37 |
| *Greater than high school* | 1.54 | 0.94 | 2.54 |  | 1.21 | 0.78 | 1.86 |  | 1.03 | 0.70 | 1.52 |
| **Socioeconomic status (ref.: I quintile, lower)** |  |  |  |  |  |  |  |  |  |  |  |
| *II* | 1.29 | 0.84 | 2.01 |  | 1.06 | 0.73 | 1.54 |  | 1.36 | 0.96 | 1.93 |
| *III* | 0.97 | 0.60 | 1.58 |  | 1.13 | 0.78 | 1.64 |  | 1.50 | 1.04 | 2.16 |
| *IV* | 1.50 | 0.90 | 2.51 |  | 1.19 | 0.75 | 1.89 |  | 1.80 | 1.15 | 2.83 |
| *V* | 1.35 | 0.74 | 2.46 |  | 1.43 | 0.80 | 2.58 |  | 2.71 | 1.55 | 4.72 |
| **Indigenous ethnicity** | 0.81 | 0.48 | 1.39 |  | 0.70 | 0.39 | 1.25 |  | 0.64 | 0.42 | 0.96 |
| **Region (ref.: north)** |  |  |  |  |  |  |  |  |  |  |  |
| *Central* | 1.37 | 0.90 | 2.09 |  | 1.23 | 0.83 | 1.83 |  | 1.33 | 0.93 | 1.90 |
| *Central-western* | 1.26 | 0.82 | 1.94 |  | 1.29 | 0.89 | 1.86 |  | 1.41 | 1.02 | 1.95 |
| *South-southeast* | 1.55 | 1.00 | 2.39 |  | 1.65 | 1.12 | 2.43 |  | 1.63 | 1.14 | 2.33 |
| **Health insurance (ref.: social security)** |  |  |  |  |  |  |  |  |  |  |  |
| *Public service* | 0.93 | 0.59 | 1.47 |  | 0.74 | 0.51 | 1.10 |  | 0.69 | 0.48 | 1.01 |
| *No coverage* | 1.02 | 0.60 | 1.73 |  | 0.89 | 0.57 | 1.39 |  | 0.91 | 0.59 | 1.41 |
