# Supplementary material for: Are overweight and obesity associated with increased risk of cesarean delivery in Mexico? A cross-sectional study from the National Survey of Health and Nutrition
Source: BMC Pregnancy Childbirth. 2019 Jul 11;19:239. doi: 10.1186/s12884-019-2393-5 (PMC6624890; doi:10.1186/s12884-019-2393-5)
Supplement: Supplementary file 3 — Table S3. Association between body mass index and cesarean delivery in multiparous women, by time elapsed between delivery and BMI measurement, Mexico, 2012. (DOCX 16 kb) [file 12884_2019_2393_MOESM3_ESM.docx]

| **Additional table 3. Association between body mass index and cesarean delivery in multiparous women, by time elapsed between delivery and BMI measurement, Mexico, 2012.** | | | | | | | | | | | |
| --- | --- | --- | --- | --- | --- | --- | --- | --- | --- | --- | --- |
|  |  |  |  |  |  |  |  |  |  |  |  |
|  |  |  |  |  |  |  |  |  |  |  |  |
|  | **< 2 years** | | |  | **2 - 4 years** | | |  | **> 4 years** | | |
| **Body mass index (ref.: normal)** | **aOR** | **[95% CI]** | |  | **aOR** | **[95% CI]** | |  | **aOR** | **[95% CI]** | |
| Overweight | 1.21 | 0.92 | 1.57 |  | 1.12 | 0.86 | 1.46 |  | 1.20 | 0.92 | 1.57 |
| Obesity | 1.80 | 1.33 | 2.44 |  | 1.66 | 1.22 | 2.25 |  | 1.75 | 1.28 | 2.40 |
| **Late antenatal care initiation** | 1.07 | 0.78 | 1.47 |  | 1.22 | 0.88 | 1.68 |  | 1.06 | 0.78 | 1.44 |
| **Five or more antenatal consultations** | 0.79 | 0.54 | 1.16 |  | 0.70 | 0.48 | 1.01 |  | 0.66 | 0.45 | 0.98 |
| **Complications during pregnancy** | 1.14 | 0.90 | 1.45 |  | 1.17 | 0.92 | 1.48 |  | 1.12 | 0.88 | 1.42 |
| **Complications at delivery** | 3.95 | 3.04 | 5.15 |  | 3.80 | 2.86 | 5.03 |  | 4.10 | 3.10 | 5.42 |
| **Diabetes Mellitus** | 1.62 | 0.67 | 3.91 |  | 1.17 | 0.51 | 2.65 |  | 1.32 | 0.56 | 3.11 |
| **Hypertension** | 0.94 | 0.61 | 1.45 |  | 1.16 | 0.78 | 1.73 |  | 1.05 | 0.68 | 1.61 |
| **Place of delivery (ref.: social security)** |  |  |  |  |  |  |  |  |  |  |  |
| *Public service facilities* | 1.03 | 0.73 | 1.44 |  | 1.19 | 0.86 | 1.66 |  | 0.99 | 0.71 | 1.38 |
| *Private facilities* | 2.39 | 1.68 | 3.39 |  | 2.75 | 1.95 | 3.87 |  | 2.12 | 1.50 | 2.98 |
| **Age at delivery (ref.: 12 - 19 years)** |  |  |  |  |  |  |  |  |  |  |  |
| 15-19 | 0.13 | 0.02 | 0.71 |  | 2.18 | 0.48 | 10.00 |  | 0.26 | 0.06 | 1.16 |
| 20-24 | 0.15 | 0.03 | 0.82 |  | 2.83 | 0.62 | 12.90 |  | 0.38 | 0.08 | 1.69 |
| 25-29 | 0.17 | 0.03 | 0.93 |  | 3.51 | 0.77 | 15.97 |  | 0.39 | 0.09 | 1.77 |
| 30-34 | 0.21 | 0.04 | 1.17 |  | 4.39 | 0.96 | 20.05 |  | 0.53 | 0.12 | 2.38 |
| 35-40 | 0.22 | 0.04 | 1.25 |  | 4.32 | 0.90 | 20.77 |  | 0.55 | 0.12 | 2.46 |
| 40-44 | 0.24 | 0.04 | 1.41 |  | 4.56 | 0.95 | 21.89 |  | 0.61 | 0.12 | 2.96 |
| +45 | 0.02 | 0.00 | 0.20 |  | 0.34 | 0.04 | 3.29 |  | 0.04 | 0.00 | 0.36 |
| **Educational level (ref.: primary or less)** |  |  |  |  |  |  |  |  |  |  |  |
| *High school* | 1.28 | 1.00 | 1.64 |  | 1.29 | 1.00 | 1.66 |  | 1.07 | 0.82 | 1.38 |
| *Greater than high school* | 1.60 | 1.18 | 2.17 |  | 1.52 | 1.11 | 2.09 |  | 1.53 | 1.12 | 2.09 |
| **Socioeconomic status (ref.: I quintile, lower)** |  |  |  |  |  |  |  |  |  |  |  |
| *II* | 1.29 | 0.96 | 1.72 |  | 1.31 | 0.97 | 1.77 |  | 1.33 | 0.99 | 1.78 |
| *III* | 1.52 | 1.08 | 2.13 |  | 1.55 | 1.11 | 2.15 |  | 1.69 | 1.22 | 2.34 |
| *IV* | 1.31 | 0.88 | 1.93 |  | 1.39 | 0.94 | 2.06 |  | 1.48 | 0.98 | 2.24 |
| *V* | 2.01 | 1.22 | 3.30 |  | 1.77 | 1.08 | 2.91 |  | 1.86 | 1.13 | 3.07 |
| **Indigenous ethnicity** | 0.66 | 0.41 | 1.06 |  | 0.75 | 0.48 | 1.16 |  | 0.65 | 0.42 | 1.01 |
| **Region (ref.: north)** |  |  |  |  |  |  |  |  |  |  |  |
| *Central* | 1.38 | 0.99 | 1.91 |  | 1.19 | 0.86 | 1.65 |  | 1.58 | 1.12 | 2.22 |
| *Central-western* | 1.25 | 0.94 | 1.66 |  | 1.13 | 0.87 | 1.48 |  | 1.31 | 0.99 | 1.75 |
| *South-southeast* | 1.25 | 0.90 | 1.73 |  | 1.08 | 0.77 | 1.53 |  | 1.19 | 0.87 | 1.64 |
| **Health insurance (ref.: social security)** |  |  |  |  |  |  |  |  |  |  |  |
| *Public service* | 0.73 | 0.53 | 1.00 |  | 0.69 | 0.50 | 0.95 |  | 0.79 | 0.57 | 1.09 |
| *No coverage* | 0.89 | 0.60 | 1.33 |  | 0.84 | 0.57 | 1.25 |  | 1.11 | 0.76 | 1.61 |
