# Supplementary material for: Are overweight and obesity associated with increased risk of cesarean delivery in Mexico? A cross-sectional study from the National Survey of Health and Nutrition
Source: BMC Pregnancy Childbirth. 2019 Jul 11;19:239. doi: 10.1186/s12884-019-2393-5 (PMC6624890; doi:10.1186/s12884-019-2393-5)
Supplement: Supplementary file 4 — Table S4. Association between body mass index (BMI) and cesarean deliveries according to parity, Mexico, 2012. (Unadjusted odds ratio). (DOCX 12 kb) [file 12884_2019_2393_MOESM4_ESM.docx]

| **Additional table 4. Association between body mass index (BMI) and cesarean deliveries according to parity, Mexico, 2012. (Unadjusted odds ratio).** | | | | | | | |
| --- | --- | --- | --- | --- | --- | --- | --- |
|  | **Nulliparous** | | |  | **Multiparous** | | |
| **Body mass index (ref.: normal)** | **OR** | **[95% CI]** | |  | **OR** | **[95% CI]** | |
|  |  |  |  |  |  |  |  |
| Overweight | 0.81 | 0.55 | 1.19 |  | 1.33 | 1.02 | 1.73 |
| Obesity | 1.59 | 0.93 | 2.72 |  | 1.91 | 1.44 | 2.55 |
